# LGBTQ+ IDENTITY SOCIAL SUPPORT AND CARE MECHANISMS AMONG LGBTQ+ MCI/ADRD CAREGIVERS

**DOI:** 10.1093/geroni/igae098.4370

**Published:** 2024-12-31

**Authors:** Nik Lampe, Ellesse-Roselee Akre, Harry Barbee, Tara McKay

**Affiliations:** University of South Florida, Tampa, Florida, United States; Johns Hopkins Bloomberg School of Public Health, Baltimore, Maryland, United States; Johns Hopkins Bloomberg School of Public Health, Baltimore, Maryland, United States; Vanderbilt University, Nashville, Tennessee, United States

## Abstract

LGBTQ+ caregivers for individuals living with mild cognitive impairment or Alzheimer’s disease and related dementias (MCI/ADRD) experience disparities in discrimination, social isolation, healthcare, and financial strain, compared to their heterosexual and cisgender counterparts. In this study, we examine the relationship between the roles of being an LGBTQ+ MCI/ADRD caregiver has on levels of LGBTQ+-identity social support and care outcomes among LGBTQ+ older adults. Using Wave 3 data (N=1,038) of The LGBTQ+ Social Networks, Aging, and Policy Study (QSNAPS), we use descriptive analyses to estimate sample characteristics and Chi-square tests and logistic regressions to test the associations between MCI/ADRD caregiver status and outcomes concerning LGBTQ+-identity social support from family, friends, coworkers, and neighbors as well as various LGBTQ+-affirming care practices. Eighty-three respondents (8.3%) in the QSNAPS Wave 3 sample identified as caregivers for individuals with neurocognitive disorders. Compared to LGBTQ+ non-MCI/ADRD caregivers, LGBTQ+ MCI/ADRD caregivers were: 51.7% less likely to have family support (p<.01) and 49.1% less likely to have neighbor support (p<.05). Compared to LGBTQ+ non-MCI/ADRD caregivers, LGBTQ+ MCI/ADRD caregivers were more likely to receive practical help from others (p< 0.1); and less likely to report their providers’ offices displaying LGBTQ-affirming materials (p< 0.1) and providers using gender-inclusive language (p< 0.1). No significant associations were found between MCI/ADRD caregiver status and other LGBTQ+-affirming care outcomes. Understanding LGBTQ+-identity social support and care mechanisms can inform targeted interventions to reduce health disparities among LGBTQ+ MCI/ADRD caregivers.

